# B cell depletion reduces T cell activation in pancreatic islets in a murine autoimmune diabetes model

**DOI:** 10.1007/s00125-018-4597-z

**Published:** 2018-03-28

**Authors:** Larissa C. Da Rosa, Joanne Boldison, Evy De Leenheer, Joanne Davies, Li Wen, F. Susan Wong

**Affiliations:** 1 Division of Infection and Immunity, Cardiff University School of Medicine, Heath Park, Cardiff, CF14 4XN UK; 2 Present Address: Department of Structural and Functional Biology, Institute of Biology, State University of Campinas, Campinas, SP Brazil; 3 Present Address: University of Sheffield, New Spring House, Sheffield, UK; 4 Section of Endocrinology, School of Medicine, Yale University, New Haven, CT USA

**Keywords:** B cell depletion, B cells, Insulitis, NOD mice, Type 1 diabetes

## Abstract

**Aims/hypothesis:**

Type 1 diabetes is a T cell-mediated autoimmune disease characterised by the destruction of beta cells in the islets of Langerhans, resulting in deficient insulin production. B cell depletion therapy has proved successful in preventing diabetes and restoring euglycaemia in animal models of diabetes, as well as in preserving beta cell function in clinical trials in the short term. We aimed to report a full characterisation of B cell kinetics post B cell depletion, with a focus on pancreatic islets.

**Methods:**

Transgenic NOD mice with a human CD20 transgene expressed on B cells were injected with an anti-CD20 depleting antibody. B cells were analysed using multivariable flow cytometry.

**Results:**

There was a 10 week delay in the onset of diabetes when comparing control and experimental groups, although the final difference in the diabetes incidence, following prolonged observation, was not statistically significant (*p* = 0.07). The co-stimulatory molecules CD80 and CD86 were reduced on stimulation of B cells during B cell depletion and repopulation. IL-10-producing regulatory B cells were not induced in repopulated B cells in the periphery, post anti-CD20 depletion. However, the early depletion of B cells had a marked effect on T cells in the local islet infiltrate. We demonstrated a lack of T cell activation, specifically with reduced CD44 expression and effector function, including IFN-γ production from both CD4^+^ and CD8^+^ T cells. These CD8^+^ T cells remained altered in the pancreatic islets long after B cell depletion and repopulation.

**Conclusions/interpretation:**

Our findings suggest that B cell depletion can have an impact on T cell regulation, inducing a durable effect that is present long after repopulation. We suggest that this local effect of reducing autoimmune T cell activity contributes to delay in the onset of autoimmune diabetes.

**Electronic supplementary material:**

The online version of this article (10.1007/s00125-018-4597-z) contains peer-reviewed but unedited supplementary material, which is available to authorised users.

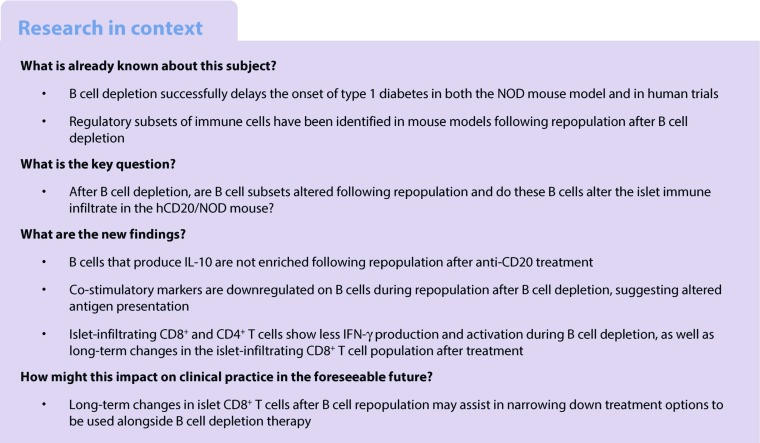



## Introduction

Type 1 diabetes, an organ-specific autoimmune disease with a multifactorial aetiology, is characterised by the immune-mediated destruction of beta cells in pancreatic islets, resulting in insufficient insulin production [1]. Although relatively few immunotherapeutic strategies delay the loss of islet beta cell function, depleting B cells using anti-CD20 monoclonal antibody (rituximab) has delayed C-peptide loss within the first year [2]. Follow-up studies demonstrated that during depletion there was a decreased antibody response to new and recall antigens [3] but that rituximab suppressed anti-insulin autoantibodies more than anti-GAD, -IA-2 and -ZnT8 autoantibodies [4]. Interestingly, during depletion, T cell proliferative responses to islet antigens increased, particularly in responders to B cell depletion therapy [5]. In addition, the frequencies of autoreactive B cells, judged by islet autoantibody-producing cell clones and by polyreactive B cells, as shown by Hep-2 cell reactivity, were unchanged a year after rituximab treatment and reconstitution [6].

In NOD mice, agents effecting timed depletion of B cells prevent diabetes [7–11] and reverse disease after onset [7, 8, 12]. These include anti-human CD20 antibody in human CD20 (hCD20)/NOD transgenic mice (in which the human gene *MS4A1*, encoding hCD20, is expressed), anti-mouse CD20 antibody, anti-CD22 antibody coupled to immunotoxin, B-Lys/BAFF neutralisation and BCMA-Fc chimerised protein [7, 8, 12]. While these strategies all influence development of diabetes, there are important differences in the effector mechanisms. The many factors determining therapeutic efficacy are not fully characterised. For example, the duration of B cell depletion may be important: monoclonal anti-CD20 that depletes B cells transiently (repopulation by 5 weeks) was not effective in preventing or protecting against diabetes [13]. Thus, it is important to understand kinetics and immunological effects following anti-B cell treatment with such agents.

In a mouse model, using a strategy and antibody similar to rituximab, responses of pathogenic CD4^+^ T cells were greater in the short term [14]. This recapitulated the finding in humans where T cell responses increased [5]. However, some potentially regulatory subsets of cells, including transitional-zone 2 (T2) B cells, T cells and Gr1^+^ cells, were increased following repopulation after depletion [15]. Thus, the protection conferred by treatment with anti-CD20 relates not only to the depletion of effector B cells but also to the increase of regulatory populations. The combination of anti-CD20 and oral administration of anti-CD3 had a synergistic effect in hCD20/NOD mice, also related to increased frequency and function of regulatory T cells [16].

In this study, we focused on characterising the effects of B cell depletion on peripheral B cells and the characteristics of the cellular infiltrate in the islets of Langerhans in the hCD20/NOD mouse. We hypothesised that B cell depletion alters the islet immune infiltrate, contributing to protection from diabetes.

## Methods

### Mice

Human CD20 (hCD20) transgenic mice on a BALB/c background (in which the human gene *MS4A1*, encoding hCD20, is expressed) [17] were backcrossed to the NOD genetic background more than ten generations, and designated hCD20/NOD mice [7]. Mice were maintained at Cardiff University in specific pathogen-free isolators or scantainers. Mice received water and food ad libitum and were housed in a 12 h dark–light cycle. Animal experiments were conducted in accordance with United Kingdom Animals (Scientific Procedures) Act, 1986 and associated guidelines.

### Diabetes incidence

Mice were monitored weekly for glycosuria (Bayer Diastix) from 12 weeks of age. Diabetes was confirmed by blood glucose levels >13.9 mmol/l.

### Anti-CD20 treatment

Female hCD20/NOD mice, aged 6–8 or 12–15 weeks were chosen at random to receive anti-hCD20 antibody (clone 2H7; Bio-XCell [West Lebanon, NH, USA]) or control IgG2b antibody (clone MPC-11; Bio-XCell [7, 14, 15]). An i.v. injection of 500 μg of antibody in 200 μl of saline solution (154 mmol/l NaCl) was followed at 3 day intervals by three i.p. injections (modified from [7]).

### Cell preparations

Pancreatic lymph nodes (PLNs) were disrupted mechanically with a 30G needle. Bone-marrow cells were flushed out from the hind legs (femur and tibia). Spleens were homogenised and erythrocytes were lysed. Pancreases were inflated with collagenase P solution (Roche, Burgess Hill, UK) in Hanks’ Balanced Salt Solution (HBSS) via the common bile duct, followed by collagenase digestion with shaking at 37°C for 10 min. Islets were isolated by Histopaque density centrifugation (Sigma-Aldrich, Gillingham, UK), hand-picked under a dissecting microscope and trypsinised to generate single-cell suspensions. Islet cells were rested at 37°C, 5% CO_2_ in complete Iscove’s Modified Dulbecco’s Medium (IMDM) overnight, before stimulation for intracellular staining.

### Flow cytometry

Single-cell suspensions were incubated with TruStain (anti-mouse CD16/32; Biolegend [London, UK]) for 10 min at 4°C, followed by fluorochrome-conjugated monoclonal antibodies against cell surface markers for 30 min at 4°C. Multivariable flow cytometry was carried out using monoclonal antibodies: CD4-FITC (GK1.5 [1:200]), CD8-PE-594 (53-6.7 [1:800]), CD103-BV510 (2E7 [1:100]), PD-1-BV785 (29F.1A12 [1:200]), IFN-γ-BV711 (XMG1.2 [1:100]), CD107a-PeCy7 (1D4B [1:200]), CD69-AF700 (H1.2F3 [1:100]), TGF-β-BV421 (TW7-16B4 [1:100]), CD38-FITC (90 [1:200]) and CD86-PeCy7 (PO3 [1:200]) (all from Biolegend); IL-10-APC (JES5-16E3 [1:200]), CD1d-BV510 (1B1 [1:200]), CD21-PE-594 (7G6 [1:1000]), CD23-BV711(B3B4 [1:800]), CD24-BV650 (M1/69 [1:400]), CD3-BV786 (145-2C11 [1:100]) and CD80-BV650 (16-10A1 [1:100]) (all from BD Biosciences, Reading, UK); CD19-efluor780 (eBio1D3 [1:800]), CD5-PeCy7 (53-7.3 [1:200]), IL-6-PerCP-Cy5.5 (MP5-20F3 [1:200]) and CD44-PerCP-Cy5.5 (IM7 [1:1600]) (all from eBiosciences, Waltham, MA, USA). All antibodies were titrated before use. Dead cells were excluded from the analysis by Live/Dead exclusion (Invitrogen, Paisley, UK). For intracellular cytokine analysis, splenocytes were either unstimulated or stimulated for 24 h with 5 μg/ml lipopolysaccharide (LPS) (Sigma-Aldrich) or 5 μg/ml anti-CD40 (Bio-XCell) and washed. After an overnight resting period, 3 h before antibody staining, phorbal 12-myristrate-13-acetate (PMA) (50 ng/ml), ionomycin (500 ng/ml) and monensin (3 μg/ml) (all from Sigma-Aldrich) were added to the cells. CD107a antibody was added prior to stimulation, as previously described [18]. Fc receptors were blocked using TruStain and, after extracellular staining, cells were fixed using fixation/permeabilisation kit (BD Biosciences) according to the manufacturer’s instructions and then stained for intracellular cytokines or appropriate isotype controls. Cell suspensions were acquired on LSRFortessa (FACSDIVA software; BD Biosciences). All analysis was performed using Flowjo software (Tree Star, Ashland, OR, USA).

### Statistical analysis

No data were excluded from the analysis. Statistical analysis was performed using GraphPad Prism version 5 (GraphPad Software, San Diego, CA, USA). For islet T cells, multivariable flow cytometric analysis was performed using SPICE (Simplified Presentation of Incredibly Complex Evaluations) version 5.1 (http://exon.niaid.nih.gov). Comparison of distributions was performed using the Mann–Whitney *U* test and a partial permutation test [19]. For diabetes incidence, the Gehan–Breslow–Wilcoxon test was used. All other data were analysed by the Mann–Whitney *U* test. 

## Results

### Kinetics of B cell subset repopulation after anti-CD20 treatment

**Fig. 1:**
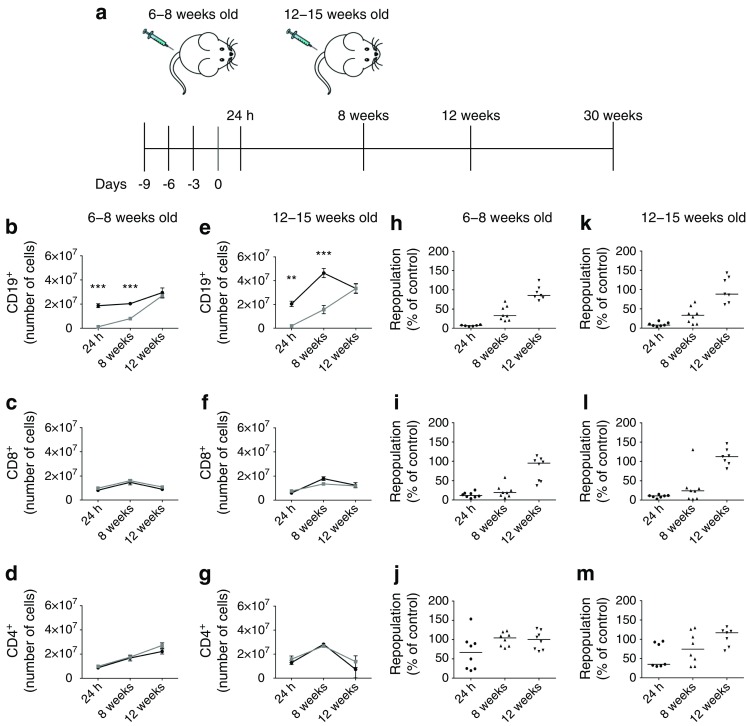
Characterisation of anti-CD20 depletion treatment. hCD20/NOD mice aged 6–8 (**b**–**d**, **h**–**j**) or 12–15 weeks (**e**–**g**, **k**–**m**) were injected with 2H7 anti-CD20 antibody (grey lines/squares in **b**–**g**) or IgG control antibody (black lines/circles in **b**–**g**). Diabetes progression was monitored and lymphocyte populations were analysed by flow cytometry at different time points after antibody depletion. (**a**) Schematic representation of injection regimen. (**b**–**g**) Cell numbers for splenic CD19^+^ B cells (**b**, **e**), CD8^+^ T cells (**c**, **f**) and CD4^+^ T cells (**d**, **g**). Data are expressed as mean ± SEM. Each time point includes a minimum of six mice from at least two independent experiments. (**h**–**m**) Percentage of B cells depleted or repopulated (calculated as individual numbers from each 2H7-treated mouse/mean number from all control antibody-treated mice) at various time points for different lymphoid organs: spleen (**h**, **k**), pancreatic lymph node (**i**, **l**), bone marrow (**j**, **m**). Horizontal lines indicate medians. **p < 0.01 and ***p < 0.001 (Mann–Whitney *U* test, control vs 2H7)

hCD20/NOD mice were treated with 2H7 or isotype control antibodies at 6–8 weeks (little insulitis) or 12–15 weeks of age (established insulitis) (Fig. 1a). We monitored disease progression in mice that were B cell-depleted at 6–8 weeks old. Diabetes was first seen in the treated mice at 29 weeks of age, delayed by 10 weeks, and incidence was reduced in the 2H7-treated groups (ESM Fig. 1). At the time of diabetes onset in the experimental group, 63% of the control mice that ultimately developed disease were diabetic, although the difference at the termination of the experiment was not statistically significant (*p* = 0.07). This delayed incidence was in keeping with our previously published observation [7]. B cells were successfully depleted in both age groups and had fully repopulated the spleen by 12 weeks post depletion (Fig. 1b, e). No change was observed in the numbers of CD4 or CD8 T cells during depletion or repopulation (Fig. 1c, d, f, g). B cell depletion in PLNs (Fig. 1i, l), vs spleen (Fig. 1h, k), was prolonged. B cells were not fully depleted in the bone marrow, compared with other lymphoid tissues (Fig. 1j, m).

### Kinetics of repopulation of B cell regulatory subsets after anti-CD20 treatment

**Fig. 2:**
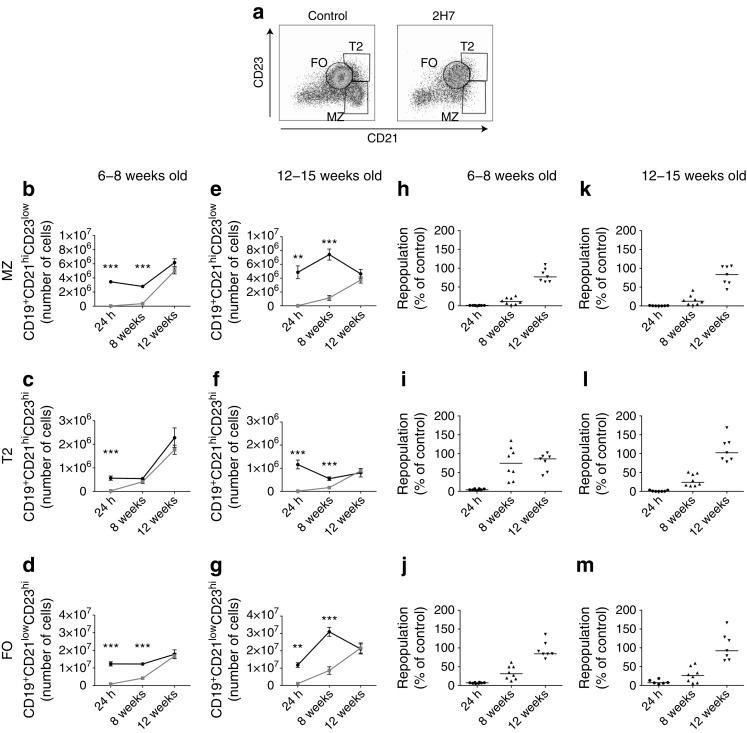
Kinetics of B cell regulatory markers after anti-CD20 antibody treatment. hCD20/NOD mice aged 6–8 weeks (**b**–**d**, **h**–**j**) or 12–15 weeks (**e**–**g**, **k**–**m**) were injected with 2H7 anti-CD20 antibody (grey lines/squares in **b**–**g**) or IgG control antibody (black lines/circles in **b**–**g**) and total splenocytes were analysed. CD19^+^ B cell populations were identified by flow cytometry at different time points after depletion. (**a**) Representative flow plots (24 h) of spleen compartments marked by CD21 and CD23 (marginal zone [MZ: CD21^hi^CD23^low^], T2 [CD21^hi^CD23^hi^]) and follicular zone [FO: CD21^low^CD23^hi^], showing flow cytometric gating of control IgG- and 2H7-treated mice (aged 6–8 weeks). (**b**–**g**) Number of B cells from MZ (**b**, **e**), T2 (**c**, **f**) and FO (**d**, **g**) spleen compartments. (**h**–**m**) Percentage of B cells depleted or repopulated for MZ (**h**, **k**), T2 (**i**, **l**) and FO (**j**, **m**) spleen compartments (calculated as individual numbers from each 2H7-treated mouse/mean number from all control antibody-treated mice). Horizontal lines indicate medians. All surface markers are shown for cells that were gated on viable CD3^−^CD19^+^. Data are expressed as mean ± SEM. Each time point includes a minimum of six mice from at least two independent experiments. **p < 0.01 and ***p < 0.001 (Mann–Whitney *U* test, control vs 2H7)

The various B cell depletion methods target different B cell zones in the spleen [7, 20]. Splenic B cell populations were mostly depleted 24 h after 2H7 treatment (Fig. 2a) and B cell numbers were significantly reduced (Fig. 2b–g). The marginal zone (Fig. 2h, k) and T2 (Fig. 2i, l), enriched in regulatory B cells (Bregs), were more successfully depleted than the follicular zone after anti-CD20 treatment (Fig. 2j, m), indicating that Bregs were not spared during depletion. Follicular zone and T2 B cells repopulated before the marginal zone. At 12 or 30 weeks after B cell depletion, there was no increase in T2 cell numbers (data not shown). This contrasts with our previous findings of increased numbers of T2 cells in older diabetic mice, which became normoglycaemic after B cell depletion with anti-CD20 antibody [7], or in normoglycaemic 30-week-old mice treated with anti-CD22 depleting antibody [8], indicating that Bregs with T2 phenotype were not enriched after B cell repopulation. These differences may be due to the use of younger and non-diabetic mice in our current study.

### B cell depletion does not enrich for B cells producing regulatory cytokines or reduce inflammatory B cells after repopulation

**Fig. 3:**
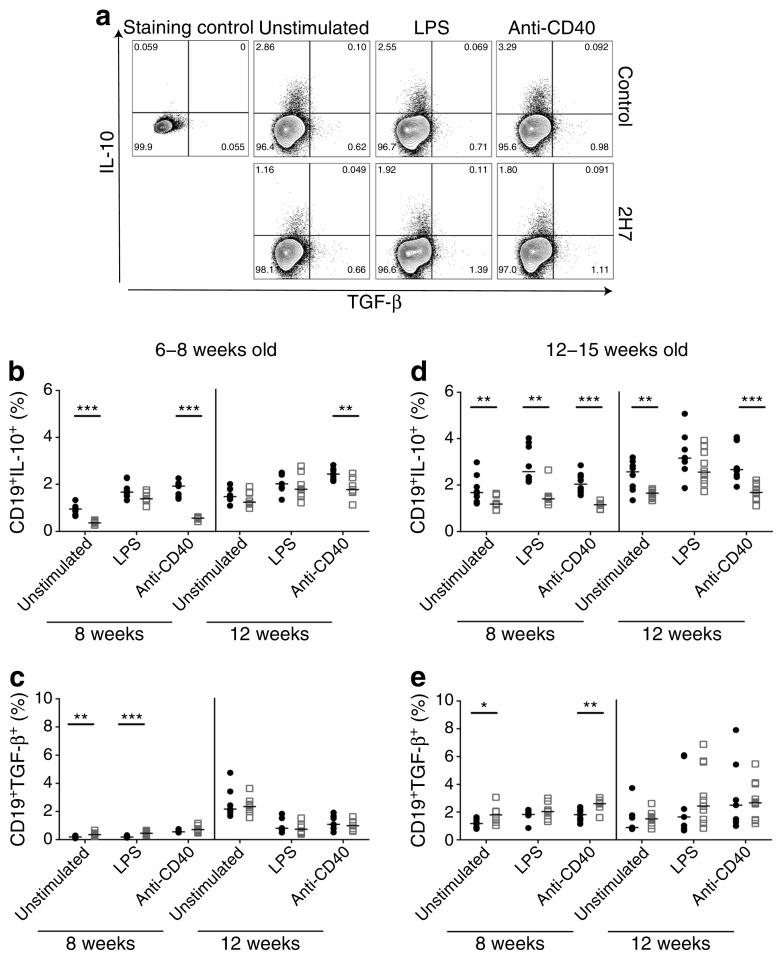
Repopulated B cells are not enriched for IL-10. B cells from the spleen were analysed for intracytoplasmic cytokines 8 and 12 weeks post treatment with control IgG (black circles) or 2H7 anti-CD20 depleting antibody (white squares) in mice aged 6–8 (**b**, **c**) or 12–15 weeks (**d**, **e**). B cells were either unstimulated or stimulated with 5 μg/ml LPS or anti-CD40. (**a**) Representative flow plots from 12 weeks post depletion. (**b**–**e**) Frequency of IL-10-producing B cells (**b**, **d**) and TGF-β-producing B cells (**c**, **e**) 8 and 12 weeks post depletion. Horizontal lines represent the medians. Each time point includes a minimum of seven mice, from at least two independent experiments. *p < 0.05, **p < 0.01 and ***p < 0.001 (Mann–Whitney *U* test, control vs 2H7)

There were significantly fewer IL-10^+^ B cells in spleens from mice treated with 2H7 vs control antibody, unstimulated or following stimulation with LPS or anti-CD40, at either 8 or 12 weeks post depletion, (Fig. 3b, d). This difference was more marked at 12 weeks, when the B cells were repopulated. Moreover, at 30 weeks post depletion, there were still fewer IL-10^+^ B cells in 2H7-treated mice, unstimulated or stimulated with anti-CD40, than in age-matched control antibody-treated mice (ESM Fig. 2). We observed no enrichment of IL-10^+^ B cells in the marginal zone or T2 compartments (data not shown) or in CD1d^hi^CD5^+^ or CD24^hi^CD38^hi^ Breg cells during or after depletion (ESM Fig. 3).

We analysed the TGF-β response, as B cell regulation can occur via TGF-β [21, 22]. In mice aged 6–8 weeks, the number of TGF-β^+^ B cells was significantly increased 8 weeks post depletion, albeit at small percentages overall (Fig. 3c, e). Differences were not maintained at 12 weeks post treatment in either of the age groups. Therefore, anti-CD20 treatment did not promote a sustained Breg phenotype either during or long after repopulation of the spleen, in either of the age groups of mice studied. We examined the proinflammatory cytokine-producing B cell populations. In the younger mice, 2H7 and control antibody treatment produced little change in the number of IL-6^+^ B cells, with or without LPS or anti-CD40 stimulation; these mice had significantly fewer IL-6^+^ B cells at 8 weeks post depletion but at 12 weeks this effect was lost and stimulation with anti-CD40 caused an increase in IL-6^+^ B cells (ESM Fig. 4).

### B cell co-stimulatory markers are downregulated during B cell depletion

**Fig. 4:**
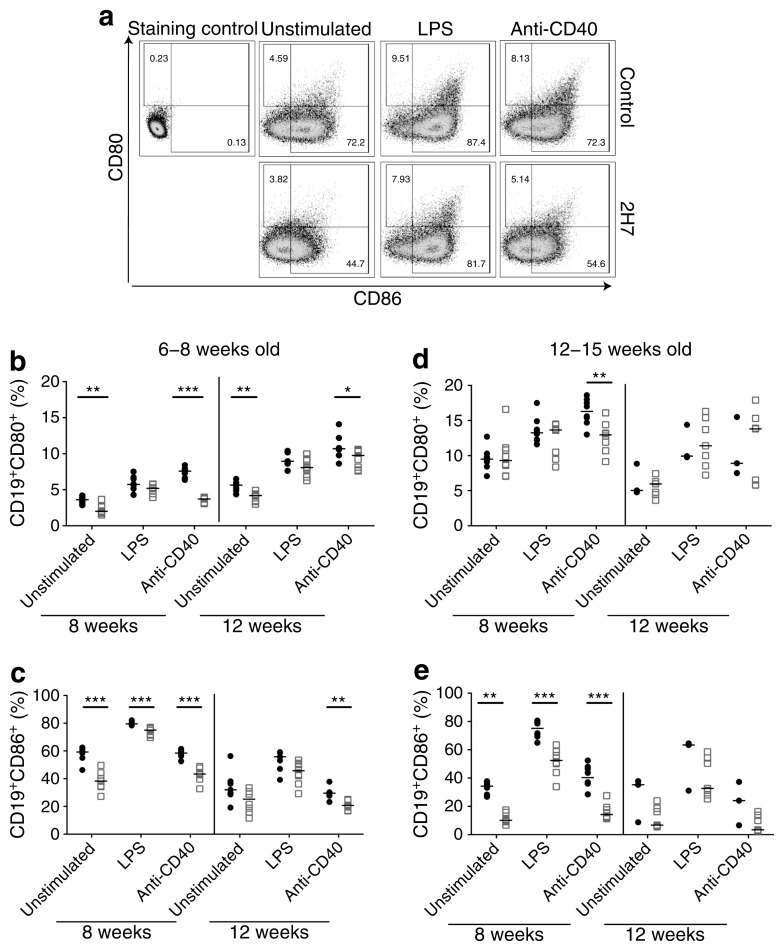
Fewer repopulated B cells express co-stimulatory molecules. Splenic B cells, from mice aged 6–8 (**b**, **c**) or 12–15 weeks (**d**, **e**) at the time of depletion, were analysed for co-stimulatory molecules 8 and 12 weeks post treatment with control IgG (black circles) or 2H7 anti-CD20 depleting antibody (white squares). The B cells were either unstimulated or were stimulated with 5 μg/ml LPS or anti-CD40 for 24 h. (**a**) Representative flow plots (12 weeks after depletion) of CD80 and CD86 expression on unstimulated or stimulated B cells. (**b**–**e**) Frequency of cells expressing CD80 (**b**, **d**) and CD86 (**c**, **e**) at 8 and 12 weeks after depletion. Horizontal lines represent the medians. Each time point includes a minimum of seven mice from at least two independent experiments. *p < 0.05, **p < 0.01 and ***p < 0.001 (Mann–Whitney *U* test, control vs 2H7)

To test the repopulating B cells for altered co-stimulatory potential, we studied the expression of CD86 and CD80 on B cells following stimulation with LPS or anti-CD40 (Fig. 4). 2H7-treated mice in both age groups expressed significantly fewer CD19^+^CD80^+^ B cells when stimulated with anti-CD40 during regeneration at 8 weeks (Fig. 4b, d); this difference was less marked at 12 weeks. More strikingly, significantly fewer B cells expressed CD86 during regeneration at 8 weeks, following the different stimuli used in both age groups of mice (Fig. 4c, e). This reduction was maintained at 12 weeks (when cells were fully repopulated), particularly following stimulation with anti-CD40, in mice aged 6–8 weeks. Unstimulated B cells from 2H7- and control antibody-treated mice at repopulation (12 weeks) were used as antigen-presenting cells in a proliferation assay to stimulate insulin-peptide-reactive CD8^+^ T cells as responders. We observed no difference in carboxyfluorescein succinimidyl ester (CFSE) dilution as a measure of proliferation of these CD8^+^ T cells (data not shown). At 30 weeks of age, whether unstimulated or stimulated, B cells expressing CD86 in both age groups were comparable, although fewer B cells expressed CD80 when stimulated with anti-CD40 in 2H7-treated mice (ESM Fig. 5).

### Effect of anti-CD20 on B cells in pancreatic islets

**Fig. 5:**
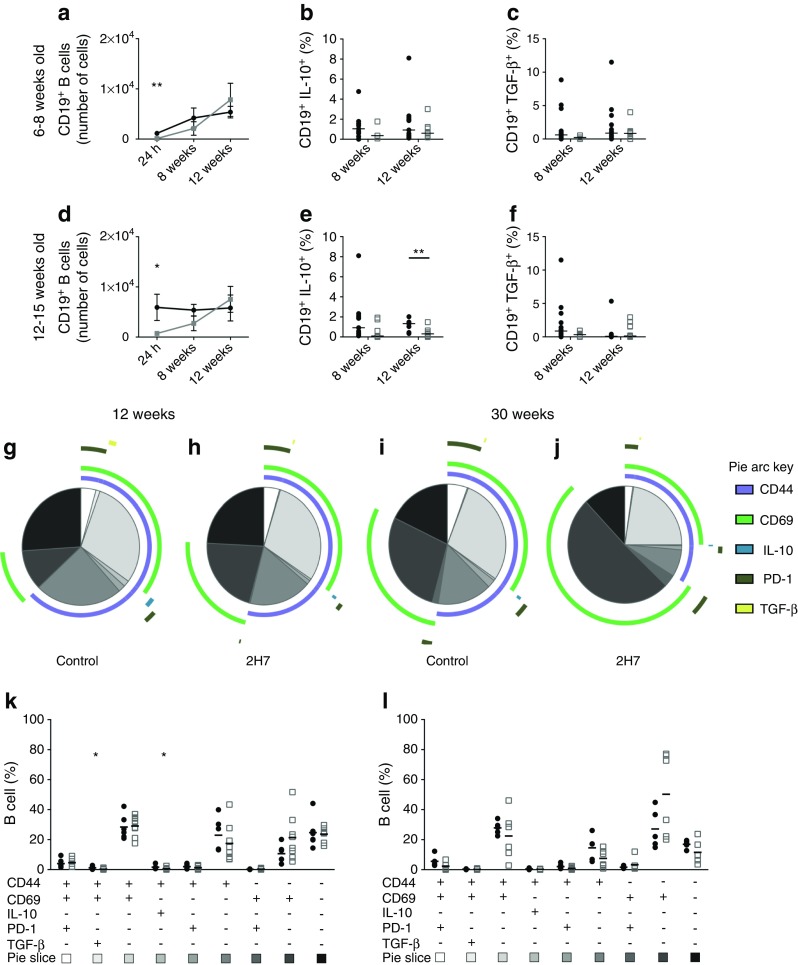
Islet-infiltrating B cells show no enrichment of regulatory cytokines. Pancreatic islets were isolated from mice aged 6–8 weeks (**a**–**c**) and 12–15 weeks (**d**–**f**) treated with control IgG (black lines/circles) or 2H7 anti-CD20 depleting antibody (grey lines/circles). (**a**, **d**) Number of CD19^+^ B cells. Data are expressed as mean ± SEM and numbers represent all islets recovered from individual pancreases. (**b**, **c**, **e**, **f**) Frequency of islet B cells stimulated with PMA/ionomycin expressing the intracellular cytokines IL-10 (**b**, **e**) and TGF-β (**c**, **f**). Horizontal lines represent medians. Black circles, control IgG; white squares, 2H7. (**g**–**l**) Multivariable analysis of B cells performed by SPICE software at 12 weeks (**g**, **h**) and 30 weeks (**i**, **j**) post depletion; (**g**, **i**) pie charts for controls; (**h**, **j**) pie charts for 2H7. Pie charts indicate different heterogeneous subsets; the coloured arcs correspond to the fraction of cells that express specific markers shown in the key. *p* = 0.2566 at 12 weeks and *p* = 0.1486 at 30 weeks (permutation test to compare the pie charts, performed by SPICE software). Graphical representations of heterogeneous subsets in pie slices are shown for 12 weeks (**k**) and 30 weeks (**l**) post depletion. Horizontal lines represent medians. Black circles, control IgG; white squares, 2H7. Cells were gated on CD8^−^CD4^−^CD19^+^. B cell combinations where the frequency did not exceed 1% are not included in SPICE analysis. Data are representative of a minimum of four mice in each group, from at least two independent experiments. *p < 0.05 and **p < 0.01 (Mann–Whitney *U* test, control vs 2H7)

We next investigated the effects of anti-CD20 on local pancreatic islet B cells during B cell depletion and repopulation in mice aged 6–8 and 12–15 weeks. Significantly fewer B cells were isolated from the pancreatic islets of anti-CD20-treated mice in both groups, as early as 24 h post antibody administration (Fig. 5a, d and ESM Fig. 6a). At 12 weeks post treatment, B cells in the islets of both groups had returned to the levels found in the control antibody-treated mice. Consistent with B cells in the spleen, a downregulation of IL-10 was observed (Fig. 5b, e). There were fewer IL-10^+^ B cells in 2H7-treated mice aged 12–15 weeks, at both 8 and 12 weeks post depletion (*p* < 0.01 at 12 weeks). Unlike splenocytes, we observed no difference in islet TGF-β^+^ B cells following 2H7 vs control antibody treatment (Fig. 5c, f). We further phenotyped islet B cells at 12 and 30 weeks after depletion in mice aged 6–8 weeks (Fig. 5g–l; for representative staining, see ESM Fig. 6b). Multivariable analysis demonstrated that, at both 12 and 30 weeks post depletion, there was no overall change in B cell phenotype or difference between the proportions of the B cell subsets. However, individual populations of IL-10^+^ and TGF-β^+^ B cells were significantly reduced 12 weeks post depletion, confirming our earlier analysis of peripheral cells. At 30 weeks post depletion, no significant difference was seen in the frequencies of the B cells within the islets, confirming that B cells had fully repopulated. However, less CD44 expression was noted, indicating lower activation.

### Anti-CD20 treatment influences T cell populations in the islet microenvironment

**Fig. 6:**
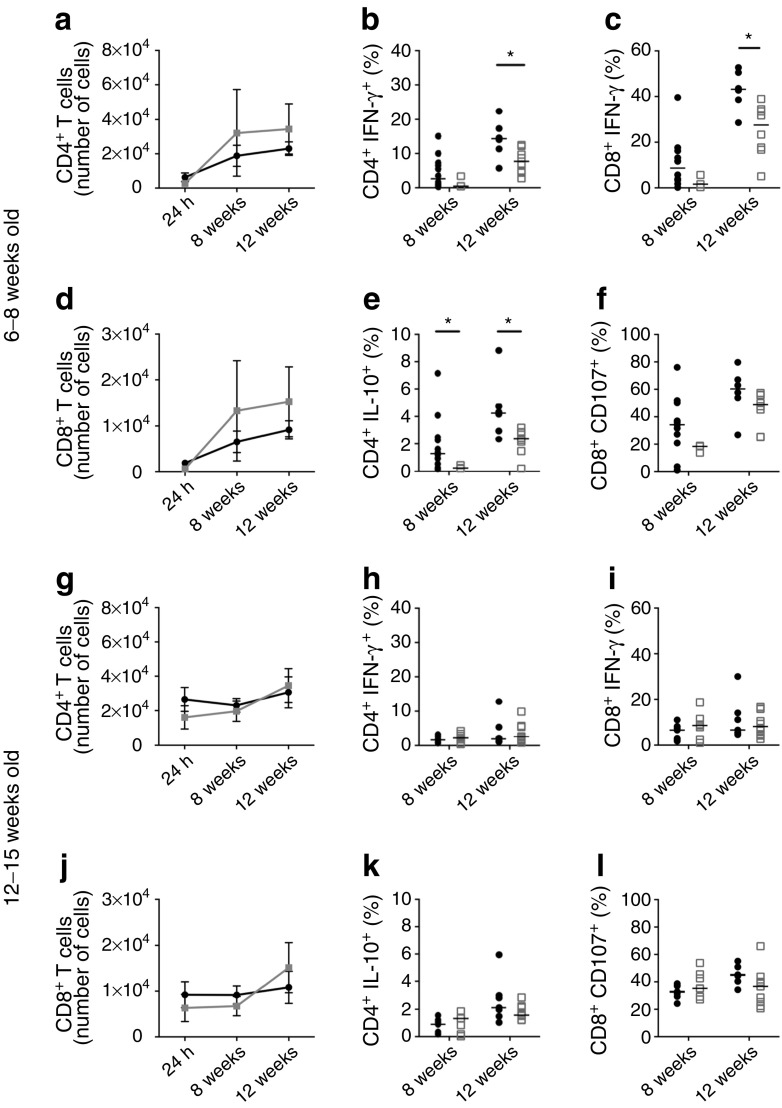
B cell depletion influences islet T cells. Pancreatic islets were isolated from mice aged 6–8 weeks (**a–f**) and 12–15 weeks (**g–l**) treated with control IgG or 2H7 anti-CD20 depleting antibody. (**a**, **g**) Number of CD4^+^ T cells and (**d**, **j**) number of CD8^+^ T cells. Each point represents cells isolated from all islets recovered from individual pancreases. Black lines/circles, control IgG; grey lines/squares, 2H7. (**b**, **e**, **h**, **k**) Cytokines in islet CD4 T cells: percentage of IFN-γ^+^ (**b**, **h**) and IL-10^+^ cells (**e**, **k**). (**c**, **f**, **i**, **l**) Cytokine and cytotoxic markers on pancreatic islet CD8 T cells: percentage of cells positive for cytokine IFN-γ (**c**, **i**) and cytotoxic CD107a (**f**, **l**). Black circles, control IgG; white squares, 2H7. Data are representative of a minimum of four mice in each group, from at least two independent experiments. Horizontal lines represent medians. *p < 0.05 (Mann–Whitney *U* test, control vs 2H7)

B cell depletion has been shown to induce peripheral regulatory T cells (Tregs) after B cell regeneration. We observed no differences in CD4^+^ or CD8^+^ T cell numbers in mouse islets following anti-CD20 vs control antibody treatment (Fig. 6a–d), at any point during depletion (24 h, 8 weeks and 12 weeks; for representative staining see ESM Fig. 7a) or at 30 weeks post depletion (data not shown). Thus, B cell depletion did not affect T cell homing to the pancreas. However, the effect of anti-CD20 treatment on CD4^+^ and CD8^+^ T cells was significantly greater in islets from mice aged 6–8 weeks vs 12–15 weeks (Fig. 6e–l). In the younger mice, during B cell depletion and regeneration (8 and 12 weeks, respectively) both IFN-γ^+^ (Fig. 6e, g) and, surprisingly, IL-10^+^ (Fig. 6f, h) CD4^+^ T cells were significantly reduced. Islet-infiltrating CD8^+^ T cells also significantly downregulated IFN-γ production during B cell regeneration at 12 weeks (Fig. 6i, k), accompanied by a decrease in CD107a expression (Fig. 6j, l) representing cytotoxic degranulation on stimulation, although this was not statistically significant. Both IFN-γ and CD107a expression in CD8^+^ T cells from 12- to 15-week-old treated mice were comparable.

### Multivariable analysis of CD4^+^ T cells revealed a shift in the local proinflammatory environment

**Fig. 7:**
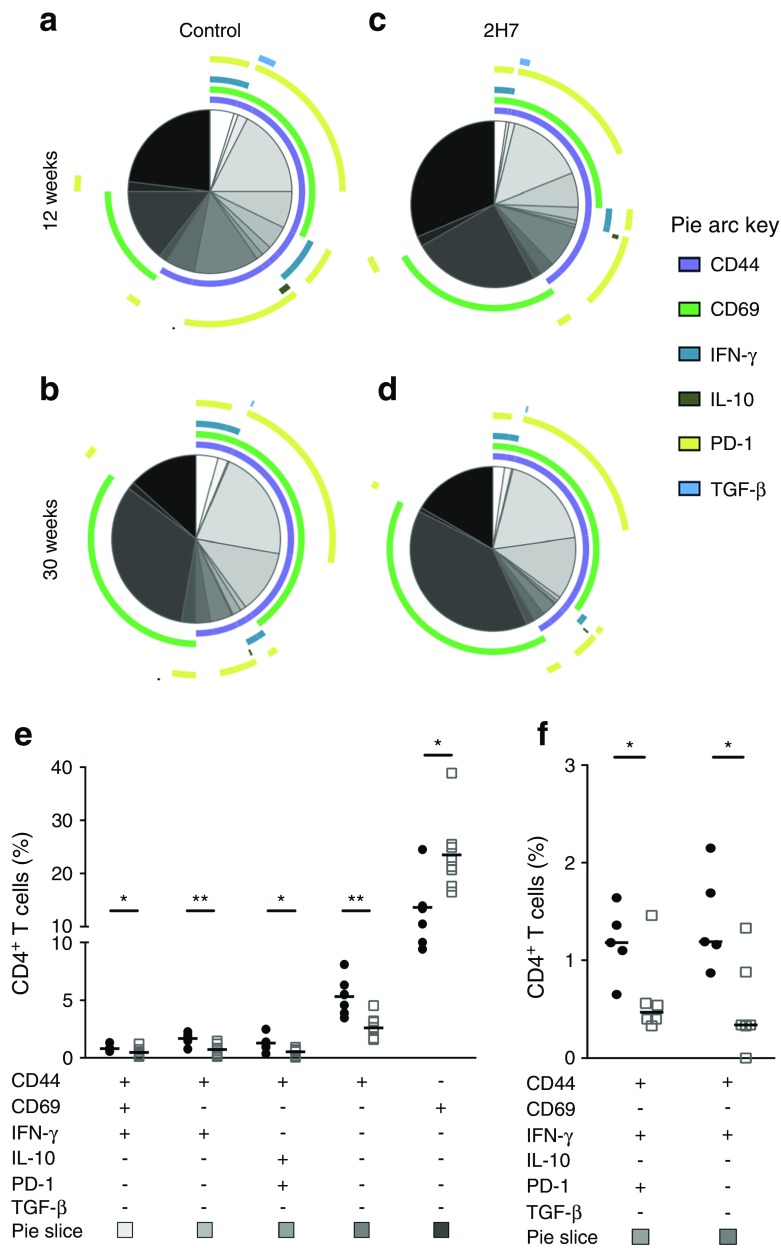
Islet CD4^+^ T cells are altered after anti-CD20 treatment. Pancreatic islets were isolated from mice aged 6–8 weeks treated with control IgG or 2H7 anti-CD20 depleting antibody. CD4^+^ T cells were analysed by flow cytometry after 12 or 27–30 weeks. Multivariable analysis of CD4^+^ T cell markers at 12 weeks (**a**, **c**) and 30 weeks post depletion (**b**, **d**) performed by SPICE software for both control IgG (**a**, **b**) and 2H7 treatment (**c**, **d**). The pie charts indicate different heterogeneous subsets, with the coloured arcs corresponding to the fraction of cells that expressed specific markers shown in the key. *p* = 0.0487 at 12 weeks; *p* = 0.4021 at 30 weeks (permutation test performed by SPICE to compare pies). Graphs illustrate the percentage of islet CD4^+^ T cell populations that were significantly changed at 12 weeks (**e**) and 30 weeks post depletion (**f**). Black circles, control IgG; white squares, 2H7. CD4^+^ T cell combinations, where the frequency did not exceed 1%, are not included in SPICE analysis. Mann–Whitney *U* test (control vs 2H7) was used to determine significance between each population. Data are representative of a minimum of six (12 weeks) or five mice (30 weeks) in each group, from at least two independent experiments. *p < 0.05, **p < 0.01

We examined activation (CD44, CD69, PD-1) and cytokine production (IFN-γ, TGF-β, IL-10) of pancreatic CD4^+^ T cells during B cell repopulation (for representative staining, see ESM Fig. 7b). Islet CD4^+^ T cells from anti-CD20 antibody-treated mice aged 6–8 weeks were examined at 12 weeks and 30 weeks after B cell depletion, when complete B cell regeneration had occurred (Fig. 7). We observed significant differences in CD4^+^ T cell composition at 12 weeks post B cell depletion (*p* < 0.05) (Fig. 7a, c), although CD4^+^ T cell subsets were comparable at 30 weeks (Fig. 7b, d). The CD4^+^ T cell subsets at 12 weeks post treatment had a less-activated phenotype, characterised by fewer CD44^+^CD4^+^ T cells, which lacked functional expression of IFN-γ; however, there was no enrichment of IL-10^+^ or TGF-β^+^CD4^+^ T cells (Fig. 7e). At 30 weeks post depletion, there were still significantly fewer IFN-γ^+^CD4^+^ T cells in 2H7-treated mice than in control IgG-treated mice (Fig. 7f). Here, the CD69 increase may be a result of altered kinetics and an increase in new CD4^+^ T cells that home to the tissue and encounter a proinflammatory environment.

### B cell depletion affects islet-associated CD8^+^ T cells long after treatment

**Fig. 8:**
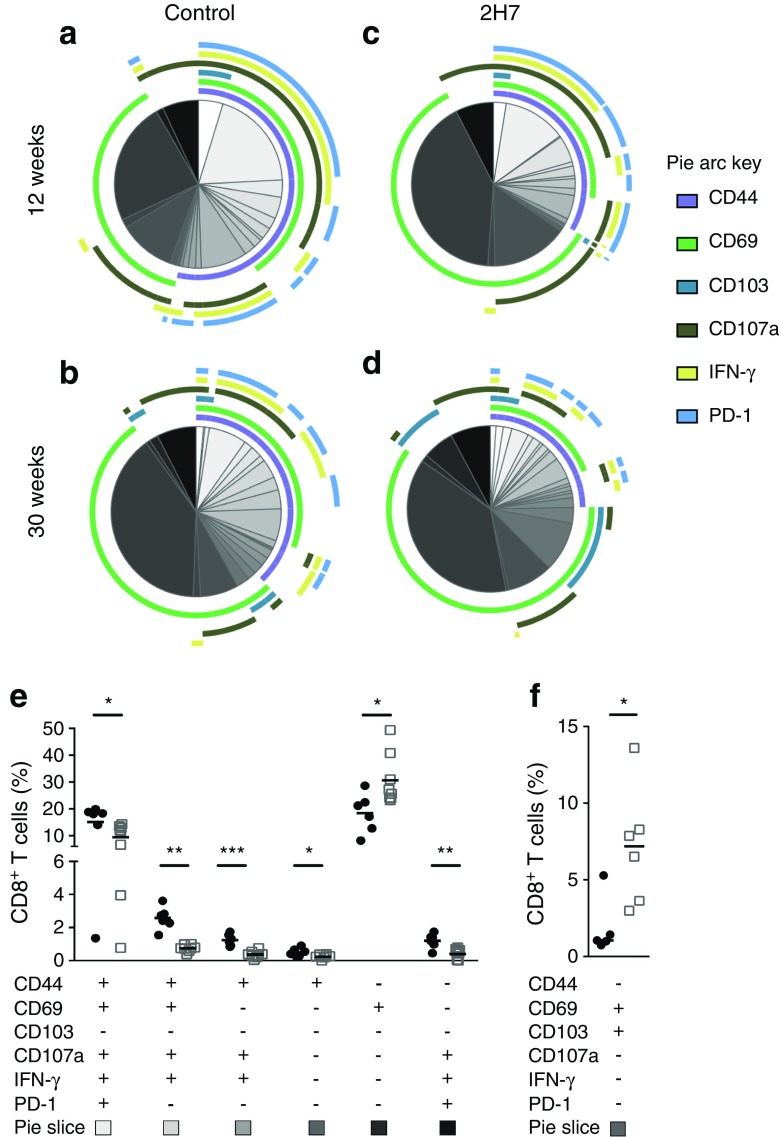
B cell depletion affects CD8^+^ T cells long after repopulation. Pancreatic islets were isolated from mice aged 6–8 weeks treated with control IgG or 2H7 anti-CD20 depleting antibody and the CD8^+^ T cells were analysed by flow cytometry 12 or 27–30 weeks later. Multivariable analysis of CD8^+^ T cell markers at 12 weeks (**a**, **c**) and 30 weeks post depletion (**b**, **d**) was performed by SPICE software for control (**a**, **b**) and 2H7 treatment (**c**, **d**). The pie charts indicate the proportion of co-expressed markers, with the coloured arcs corresponding to the fraction of cells that expressed specific markers, shown in the key. *p* = 0.0408 at 12 weeks; *p* = 0.5680 at 30 weeks (permutation test performed by SPICE to compare pie slices). Graphs illustrate the percentage of individual population of CD8^+^ T cells, expressing different surface markers and cytokines, that were significantly changed at 12 weeks post depletion (**e**) and the percentage of the individual population of CD69^+^CD103^+^CD8^+^ T cells significantly changed at 30 weeks post depletion (**f**). Black circles, control IgG; white squares, 2H7. CD8^+^ T cell combinations where the frequency did not exceed 1% are not included in the SPICE analysis. Mann–Whitney *U* test (control vs 2H7) was used to determine significance between each population. Data are representative of a minimum of six (12 weeks) or five (30 weeks) in each group, from at least two independent experiments. *p < 0.05, **p < 0.01 and ***p < 0.001

We next analysed the impact of B cell depletion on local CD8^+^ T cells in the islets of hCD20/NOD mice aged 6–8 weeks, focusing on the islet environment during repopulation (Fig. 8; see ESM Fig. 8 for representative staining). Particularly striking, was the high influx of heterogeneous CD8^+^ T cell subsets into the pancreatic islets after initial damage has occurred [23, 24]. At 12 weeks post depletion, islet-infiltrating CD8^+^ T cells significantly differed in their composition (Fig. 8a, c) and were characterised by a lack of effector function typified by less CD44 expression coupled with decreased IFN-γ and CD107a production, confirming our earlier observations (Fig. 8e) (*p* < 0.05). As with islet CD4^+^ T cells, we observed an increase in CD69^+^CD8^+^ T cells in the 2H7-treated mice, which may represent naive CD8^+^ T cell subsets being activated locally.

At 30 weeks post treatment, islet CD8^+^ T cell subsets were comparable; although there were fewer activated CD8^+^ T cells, this was not statistically significant (Fig. 8b, d). Interestingly, CD8^+^ T cells expressing CD103 and CD69, which indicate a tissue-resident memory (T_RM_) CD8 T cell phenotype and are associated with controlling tissue immunity after infection [25], were enriched in the islets of 2H7-treated mice (Fig. 8f).

## Discussion

In the current study, we observed changes in the inflammatory islet environment during B cell regeneration and these persisted long after anti-CD20 treatment. We demonstrated a significant decrease in effector function of CD4^+^ and CD8^+^ T cells, with a reduction in IFN-γ production, correlated with a downregulation of activation markers. These changes were more pronounced in mice aged 6–8 weeks vs 12–15 weeks, possibly reflecting the difference in lymphocyte behaviour recently demonstrated in islets in NOD mice before and after insulitis [26]. B cell depletion also reduced the expression of CD80 and CD86 on peripheral B cells when stimulated during the B cell regeneration period in the hCD20/NOD mouse model. Moreover, B cells with a regulatory phenotype were depleted in the periphery and were not increased following regeneration.

As reported previously, we demonstrated that anti-CD20 treatment, in this transgenic system, successfully targets the B cells in peripheral and local tissue [7]. Although all B cell populations were depleted, transitional- and marginal-zone B cells were more susceptible (not due to different hCD20 expression, data not shown). Our work supports some previous findings [5] but differs from other work indicating that the follicular zone was targeted more readily [20, 27]. This may be due to different mouse genetic background [27] and the different monoclonal antibody used [20]. Our data, consonant with other studies, demonstrated the depletion of regulatory B cell populations in the marginal zone and T2 compartments, CD1d^hi^CD5^+^ and CD24^hi^CD38^hi^ subsets, along with other B cells (data not shown). B cells with regulatory potential were not spared after B cell depletion. However, TGF-β^+^ B cells were proportionally significantly increased during depletion, although the percentages were small, whereas IL-10 production from B cells was not enriched during B cell regeneration under our experimental conditions. Furthermore, Breg subsets were not enriched during regeneration. This corroborated our earlier findings in the BDC2.5 transgenic mouse model, showing that CD1d^−^ B cells are more protective than CD1d^+^ B cells, dependent on cell–cell contact, but not IL-10 [14]. Therefore, B cells may confer protection after B cell depletion, but not via a typical Breg IL-10-mediated mechanism, even though overall anti-CD20 depletion, including depletion of the IL-10-producing B cells, increases the T cell activation observed immediately after B cell depletion [5, 14]. However, B cells located in the peritoneum are spared from depletion [28] and it is known that these cells (B-1) can produce IL-10 [29]. We did not examine peritoneal B cells, so the ability of these cells to contribute to regulation of the immune response cannot be ruled out.

We observed a reduction in both CD86 and CD80 co-stimulatory molecules upon stimulation, more strikingly with anti-CD40, during repopulation of B cells in 2H7-treated mice. Lack of CD86 expression can impair T cell activation, specifically in NOD mice [30]. Others have shown that CD80/86 expression on B cells is essential for activating proteoglycan-specific autoreactive T cells in an arthritic mouse model [31]. Anti-CD20 B cell depletion influenced the immunostimulatory environment in the secondary lymphoid organs, marked by a reduced expression of CD86/CD80 along with MHC class II, in a marmoset model of autoimmune encephalomyelitis [32]. When we examined regenerated B cells after full repopulation (12 weeks after depletion), we found no functional difference in the ability of the B cells from 2H7-treated mice to present insulin-specific peptide to insulin-specific CD8^+^ T cells in the proliferative assay that we employed (data not shown). However, because cells were not tested at an earlier time point during regeneration, we cannot discount the possibility that lack of co-stimulatory molecules alters antigen presentation in vivo during the regeneration period. This is indicated, indirectly, by the effects observed in the islets.

In human clinical trials, beta cell function is temporarily preserved after B cell depletion [33] but the effects on pancreatic immune cell infiltrate during B cell repopulation cannot be studied. Our current study showed a 10 week delay in onset of diabetes, although following extended observation, the endpoint was not statistically significant, in keeping with the human observations that B cell depletion delays disease progression. Our current study gives an important insight into the treatment effects on pancreatic islets, which are ultimately the target of protection. Interestingly, we showed no enrichment of IL-10 or TGF-β from B cells in the islets of anti-CD20-treated mice. The IL-10^+^ B cell population was downregulated at 12 weeks post treatment but was comparable with that in the control mice long after repopulation. Overall, the regulatory phenotype of B cells in islets was not altered by anti-CD20 treatment. Previously, it has been reported that islet B cells become CD20^−^CD138^+^ plasma cells after anti-CD20 treatment [20]. While murine CD20 expression was not studied here, we demonstrated significant B cell depletion in 12- to 15-week-old mice, which have established insulitis. CD86/80 expression has been described in the islet B cells previously, along with the production of TNF-α [34]. Though not specifically addressing the levels of CD80/CD86 or TNF-α on islet B cells after anti-CD20 treatment, we did not observe any IFN-γ production from either control IgG- or 2H7-treated mice (data not shown).

B cell depletion affects T cell regulation [5, 14, 35]. No obvious differences in total T cells were observed, supporting the human anti-CD20 depletion studies [5]. However, we did demonstrate that both effector CD4^+^ and CD8^+^ islet-infiltrating T cells were downregulated, including inflammatory IFN-γ production. This supports the notion that B cell depletion modulates T cell responses [5, 14]. While regulatory T cells are enriched during B cell repopulation [7, 8], we did not observe enrichment of IL-10 or TGF-β in islet-infiltrating cells; in fact IL-10^+^CD4^+^ T cells were downregulated. Thus, local Tregs may operate through IL-10- and TGF-β-independent mechanisms. In mice, peripheral T cells have decreased effector cytokines [8] and macrophages from 2H7-treated mice do not present antigen to T cells as efficiently as untreated macrophages [7]. Furthermore, CD11b^+^GR1^+^ myeloid-derived suppressor cells are induced post B cell depletion, dependent on a cell–cell contact mechanism independent of IL-10 [15]. Here, we show that B cell depletion directly affects the T cell composition of the islet infiltrate, long after the repopulation of B cells, possibly as a result of B cell–macrophage or B cell–dendritic cell crosstalk occurring directly in the pancreas.

We speculate that other mechanisms may play a role in the pancreatic islets. CD44 is an important mediator in inflammation [36] and anti-CD44 antibody successfully delays the onset of diabetes in the NOD mouse [37]. Long after B cell depletion, decreased CD44 expression on CD8^+^ T cells may contribute to the protection seen in B cell-depleted animals, especially as the ligand for CD44 (hyaluronic acid) is expressed in islets during inflammation [37]. Furthermore, we observed an increase in CD69^+^CD103^+^CD8^+^ T cells, which may be a regulatory CD8^+^ T cell population in the islets, long after antibody treatment and B cell repopulation. This specialised CD8^+^ T cell (T_RM_) population, found in tissues after viral infections, can control local immunity [38]. It is possible that these cells may also play a role in inflammatory disease [25] and autoimmunity. Interestingly, this T_RM_ population has recently been identified in individuals newly diagnosed with type 1 diabetes [39].

Our data is consonant with findings made in a small number of participants in the TrialNet study, showing maintenance of increased frequencies of autoreactive and polyreactive B cells before and after anti-B cell therapy (1 year) [6]. Thus, therapeutic efficacy for the limited time studied was not related to maintaining depletion of autoreactive B cells or increase in regulatory B cell subsets. Anti-B cell therapy with rituximab is one of the few immunotherapeutic strategies trialled thus far in humans that has shown transient efficacy in delaying the decline of C-peptide, along with anti-CD3, anti-LFA1 and CTLA4-Ig [40–43]. It is likely that more than one therapeutic strategy will be required for effective immunotherapy of type 1 diabetes. Ideally, this would encompass agents with differing mechanisms to complement each other (e.g. depletion of autoreactive lymphocytes, while increasing endogenous regulation). Our results, indicating that islet-targeting CD8^+^ T cells may be more affected by the B cell treatment, suggest that a potential adjunct therapy to rituximab could be one that maintains these changes in CD8^+^ T cells.

In conclusion, we show potential new mechanisms in the local tissue that may contribute to delay in diabetes onset following B cell depletion therapy at an early age. Further investigation is ongoing to dissect these mechanisms, both during and after B cell depletion. However, a key point is that removal of B cells alters effector T cells, either directly or through an antigen-presenting cell population, in the pancreatic islets.

## Electronic supplementary material

ESM Figs (PDF 1.96 mb)

## Data Availability

The datasets generated and/or analysed during the current study are available from the corresponding author on reasonable request.

## Abbreviations

Breg: Regulatory B cell
hCD20: Human CD20
LPS: Lipopolysaccharide
PLN: Pancreatic lymph node
PMA: Phorbal 12-myristrate-13-acetate
SPICE: Simplified presentation of incredibly complex evaluations
T2: Transitional 2
Treg: Regulatory T cell
T_RM_: Tissue-resident memory
