# Supplementary material for: B cell depletion reduces T cell activation in pancreatic islets in a murine autoimmune diabetes model
Source: Diabetologia. 2018 Mar 28;61(6):1397–410. doi: 10.1007/s00125-018-4597-z (PMC6449006; doi:10.1007/s00125-018-4597-z)
Supplement: Supplementary file 1 — (PDF 1.96 mb) [file 125_2018_4597_MOESM1_ESM.pdf]

ESM Fig. 1

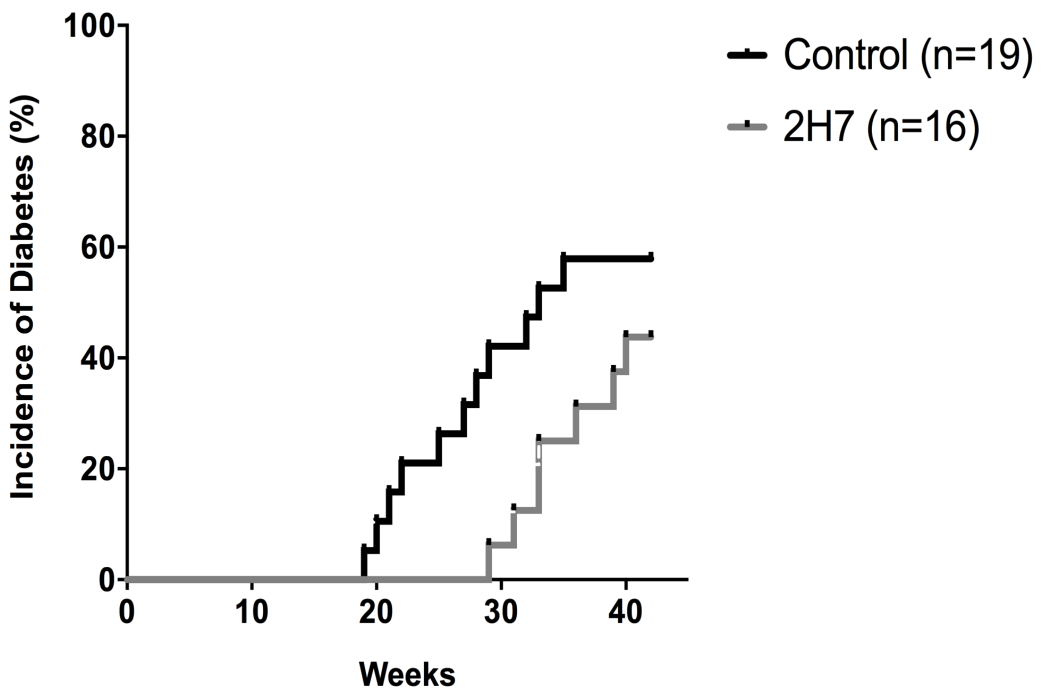

ESM Fig. 1. B cell depletion delays onset of diabetes in hCD20/NOD mice. Groups of hCD20/NOD mice were injected with control IgG or 2H7 antibody at 6-8 weeks of age. Diabetes was confirmed by blood glucose levels greater than 13.9mmol/L. Graph represents 19 mice in the control group and 16 mice in the 2H7-treated group, showing that all mice in the 2H7-treated group had a non-statistically significant trend towards delay in diabetes onset (p= 0.0758).

ESM Fig.2

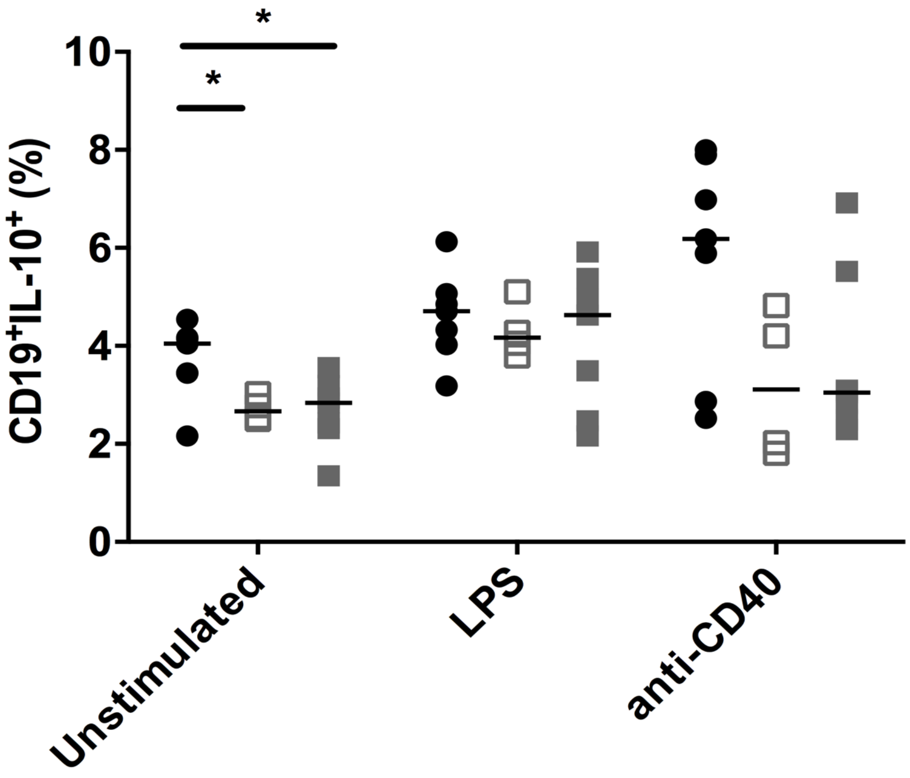

ESM Fig. 2. Splenic B cells from 2H7-treated mice at 30 weeks show no IL-10 enrichment. (a) Splenic B cells, from mice aged either 6-8 or 12-15-weeks old at the time of B cell depletion, with control IgG (black circles) or 2H7-depleting antibody (open grey squares aged 6-8 weeks, filled grey squares aged 12-15 weeks), were examined 30 weeks post-treatment. The B cells were either unstimulated, or stimulated with 5μg/ml LPS or anti-CD40 and the frequency of IL-10+ B cells is shown. Black line represents the median. Each time point includes a minimum of 4 mice. *p<0.05 (Mann Whitney U test).

ESM Fig. 3

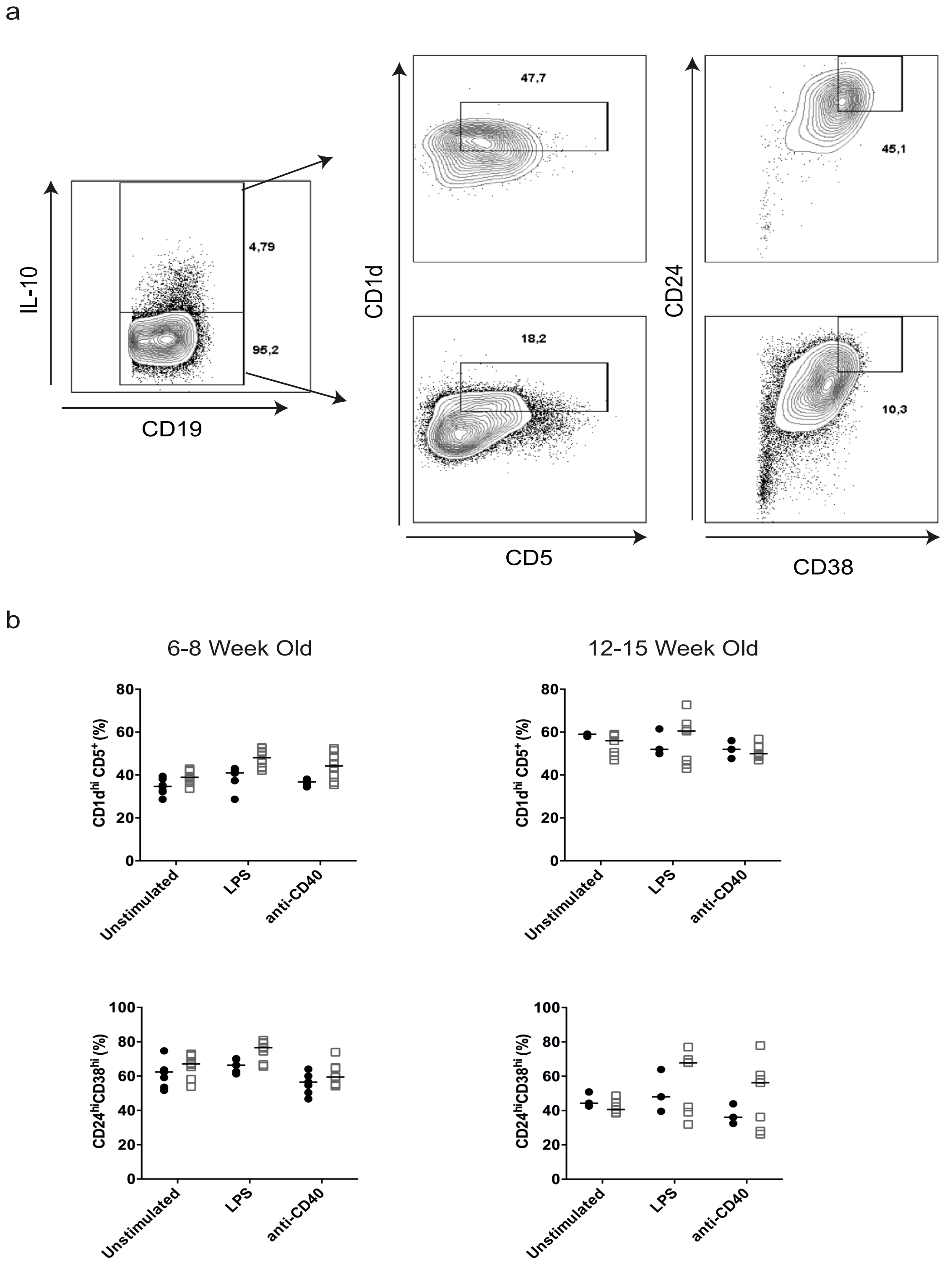

ESM Fig. 3. IL-10+ B cells are not enriched for Breg markers. Splenic B cells, from mice aged either 6-8 or 12-15-weeks old at the time of B cell depletion, treated with control IgG or 2H7-depleting antibody, were examined 12 weeks post-treatment. The B cells were either unstimulated, or stimulated with 5 μg/ml LPS or anti-CD40. (a) Representative flow plots showing IL-10+ B cell expression of CD1dhiCD5+ (top) and CD38hiCD24hi populations (bottom). (b) Graphs show the percentage of IL-10+ producing B cells expressing CD1dhiCD5+ and CD38hiCD24hi in both age groups, in either unstimulated or stimulated treatment groups. Control, black circles; 2H7, grey squares. Black line represents the median. Data are representative of at least 2 independent experiments, 6-8-weeks old with a minimum of 7 mice and 12-15-weeks old with a minimum of 3 mice.

ESM Fig. 4

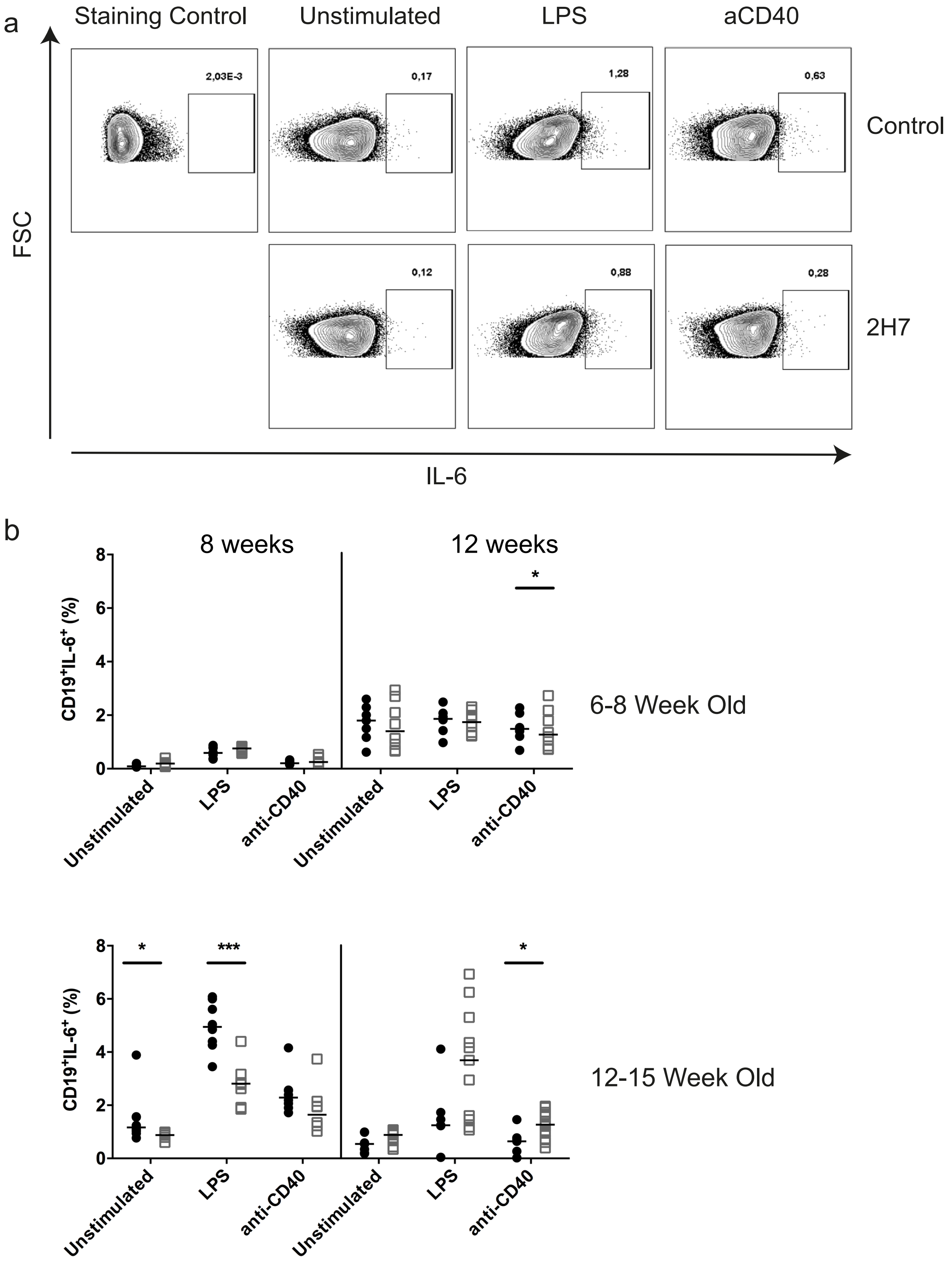

ESM Fig .4. Splenic B cells from 2H7-treated mice do not produce less IL-6. B cells from the spleen were analysed for intra-cytoplasmic cytokine IL-6, 8 and 12 weeks post-treatment with control IgG (black circles) or 2H7 depleting antibody (grey squares), administered to mice aged either 6-8 or 12-15-weeks old. The B cells were unstimulated, or stimulated with 5μg/ml LPS or anti-CD40. (a) Representative flow plots show IL-6 production from control IgG or 2H7-treated mice (b) Percentages of IL-6-producing B cells from 6-8-week-old treated mice (top) and 12-15-week-old treated mice (bottom). Black line represents the median. Each time point includes a minimum of 7 mice, from at least 2 independent experiments. ***$p<0.001$, *$p<0.05$ (Mann Whitney U test).

ESM Fig. 5

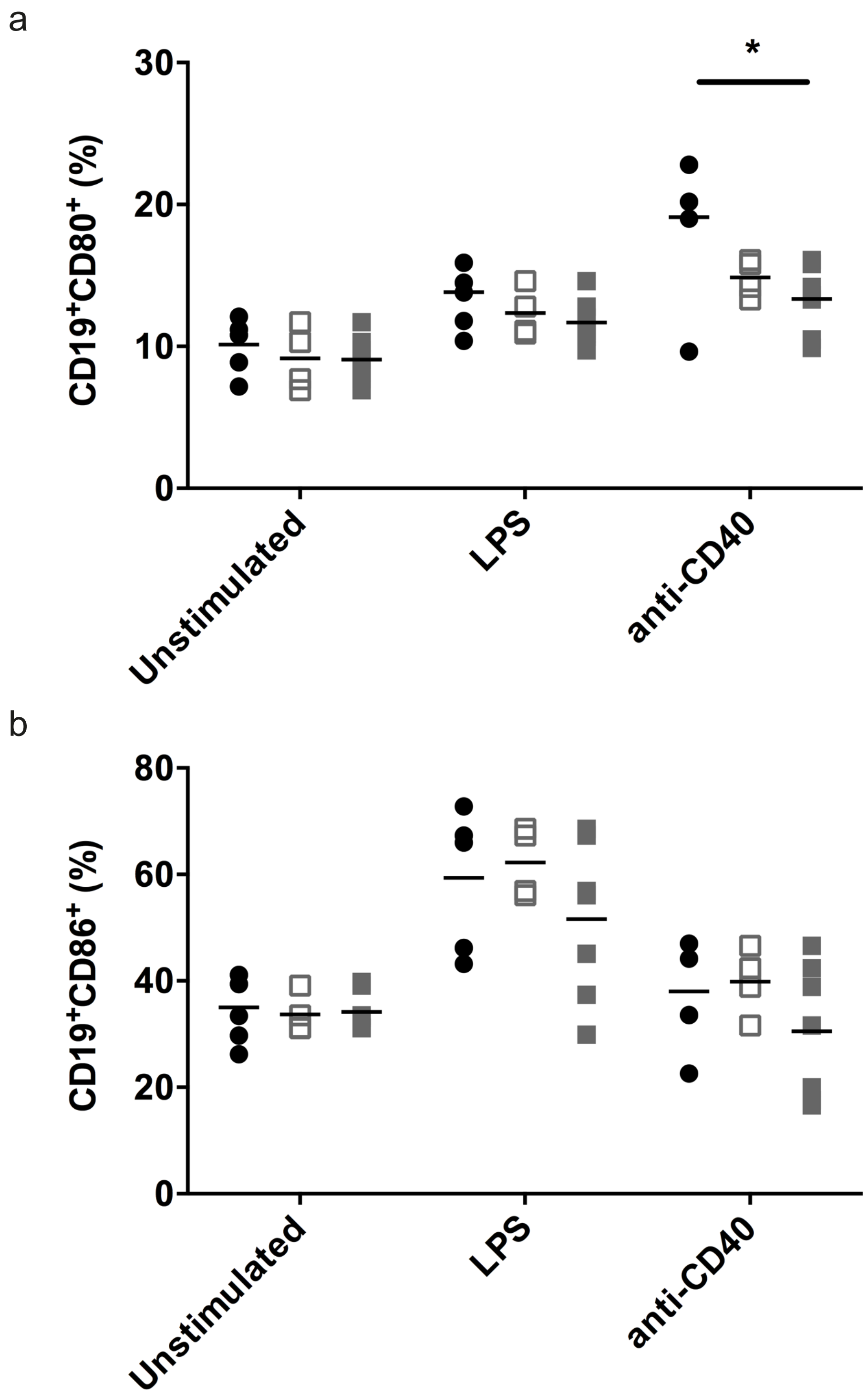

ESM Fig. 5. Splenic B cells from 2H7-treated mice at 30 weeks have comparable expression of co-stimulatory molecules. Splenic B cells, from mice aged either 6-8 or 12-15-weeks old at the time of B cell depletion, were analysed for co-stimulatory molecule expression, 30 weeks post-treatment with control IgG (black circles) or 2H7-depleting antibody (open grey squares aged 6-8 weeks, filled grey squares aged 12-15 weeks). The B cells were either left unstimulated or stimulated with 5μg/ml LPS or anti-CD40. (a) Frequency of CD80 expression and (b) CD86 expression. Black line represents the median. Each time point includes a minimum of 4 mice. *$p<0.05$ (Mann Whitney U test).

ESM Fig. 6

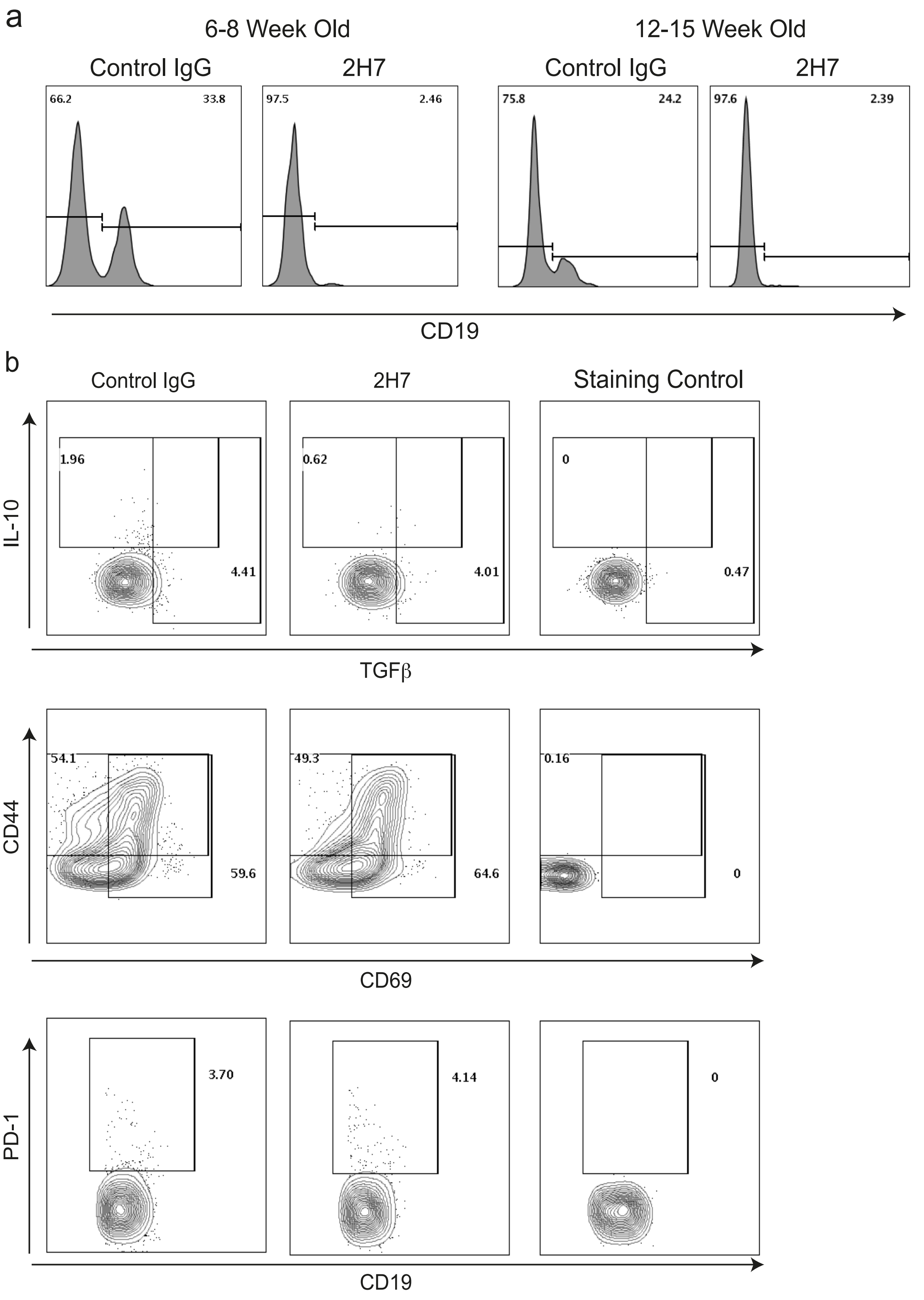

ESM Fig 6. Representative flow plots for islet-infiltrating B cells. Pancreatic islets were isolated from mice, treated with control IgG or 2H7 depleting antibody, at ages 6-8-weeks old or 12-15-weeks old. (a) Representative histograms show B cells in pancreatic islets, 24hrs after injection regimen, from control IgG-treated animals and 2H7-treated animals. (b) Representative flow plots showing IL-10 and TGF-β cytokine staining (top), CD44 and CD69 surface markers (middle) and PD-1 surface marker (bottom) on islet B cells, 12 weeks after control IgG or 2H7 treatment. Staining controls represent islet cells stained with only live/dead viability dye, CD4, CD8 and CD19 antibodies, and gated on live CD4- CD8- CD19+ B cells.

ESM Fig. 7

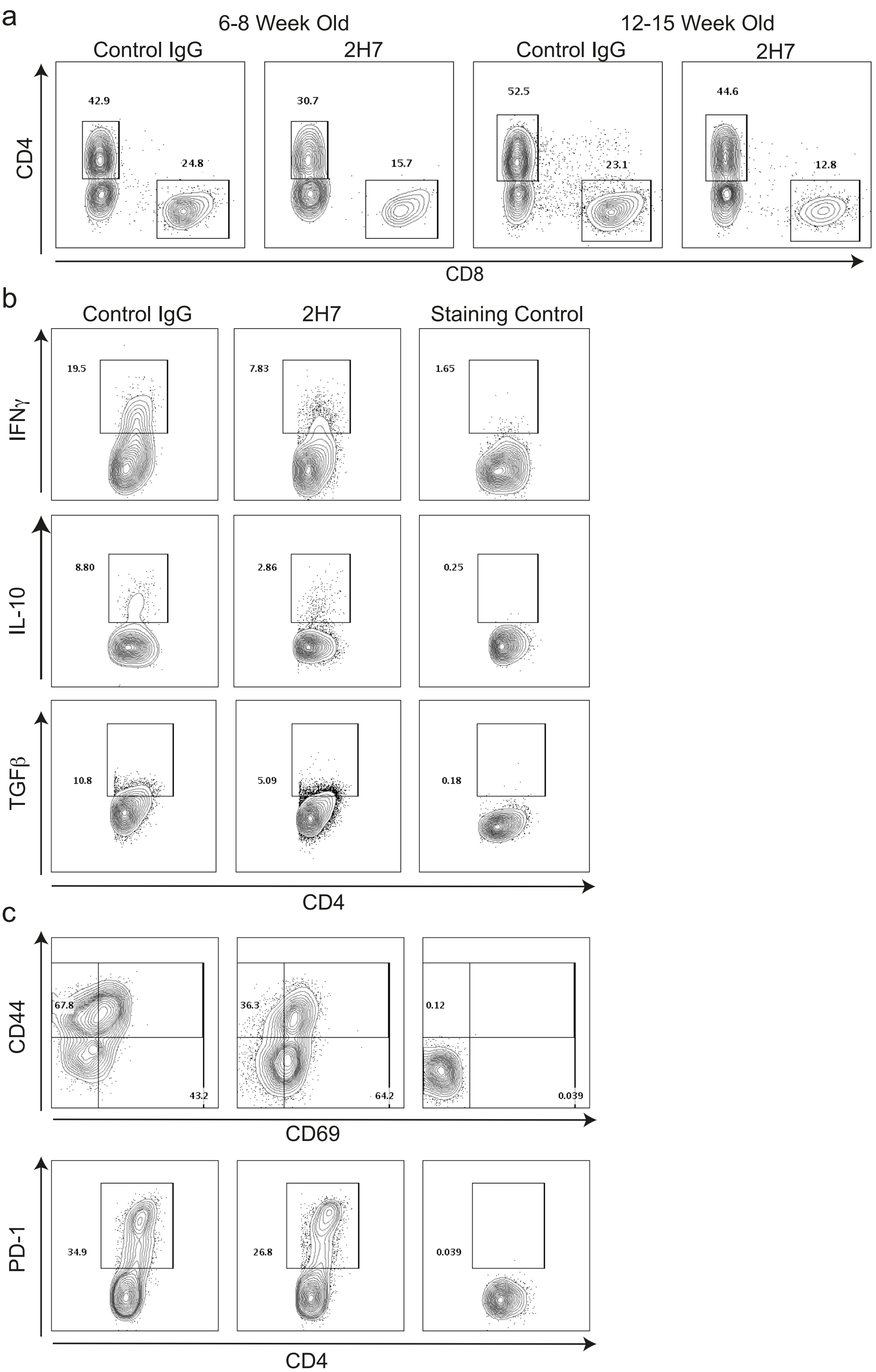

ESM Fig.7. Representative flow plots for islet-infiltrating T cells. Pancreatic islets were isolated from mice, treated with control IgG or 2H7 depleting antibody, at ages 6-8-weeks old or 12-15-weeks old. (A) Representative flow plots showing both CD4 and CD8 T cells in pancreatic islets, 24hrs after injection. (B) Representative flow plots for cytokine staining on CD4 T cells 12 weeks after B cell depletion, illustrating IFN-γ (top), IL-10 (middle) and TGF-β (bottom). (C) Representative flow plots showing CD44 and CD69 (top) and PD-1 (bottom) surface markers on CD4 T cells. Staining controls represent islet cells stained with only live/dead viability dye, CD4, CD8 and CD19 antibodies, and gated on live CD4+ CD8- CD19- CD4 T cells.

ESM Fig. 8

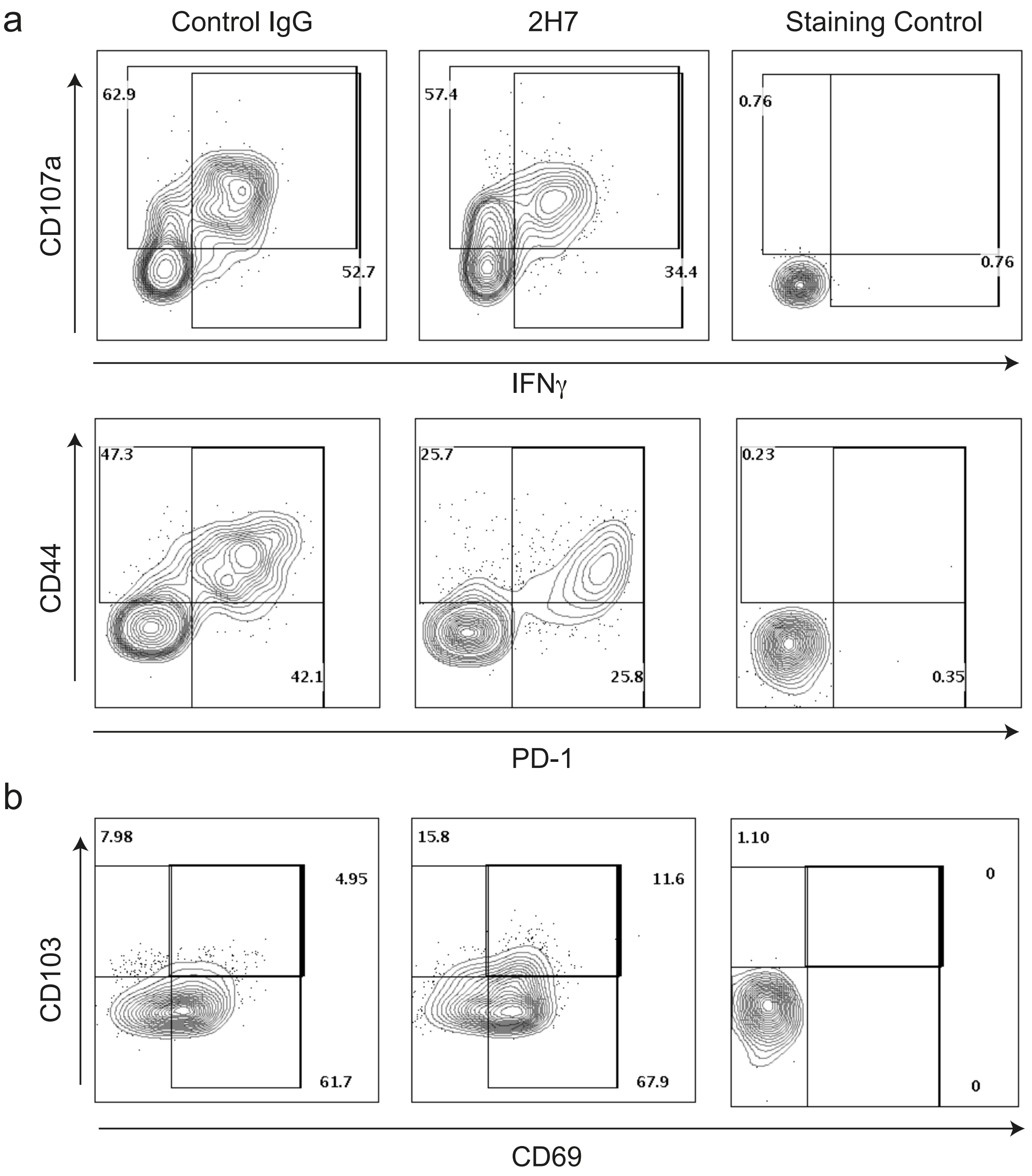

ESM Fig. 8. Representative flow plots for islet-infiltrating CD8 T cells. Pancreatic islets were isolated from mice, treated with control IgG or 2H7 depleting antibody, at age 6-8-weeks old. (A) Representative flow plots showing cytotoxic and cytokine markers CD107a and IFN-γ respectively (top) and CD44 and PD-1 (bottom) surface markers, 12 weeks after injection. (B) Representative flow plots showing CD103 and CD69 expression on CD8 T cells, 30 weeks after treatment. Staining controls represent islet cells stained with only live/dead viability dye, CD4, CD8 and CD19 antibodies, and gated on live CD4- CD8+ CD19- CD8 T cells.
